# A Tree Based Broadcast Scheme for (*m*, *k*)-firm Real-Time Stream in Wireless Sensor Networks

**DOI:** 10.3390/s17112578

**Published:** 2017-11-09

**Authors:** HoSung Park, Beom-Su Kim, Kyong Hoon Kim, Babar Shah, Ki-Il Kim

**Affiliations:** 1 Department of Informatics, Gyeongsang National University, Jinju 52828, Korea; hspark0865@gmail.com (H.P.); bumsou10@gnu.ac.kr (B.-S.K.); khkim@gnu.ac.kr (K.H.K.); 2 College of Technological Innovation, Zayed University, Dubai 19282, UAE; babar.shah@zu.ac.ae; 3 Department of Computer Science and Engineering, Chungnam National University, Daejeon 34134, Korea

**Keywords:** (*m*, *k*)-firm real-time, broadcast, wireless sensor networks

## Abstract

Recently, various unicast routing protocols have been proposed to deliver measured data from the sensor node to the sink node within the predetermined deadline in wireless sensor networks. In parallel with their approaches, some applications demand the specific service, which is based on broadcast to all nodes within the deadline, the feasible real-time traffic model and improvements in energy efficiency. However, current protocols based on either flooding or one-to-one unicast cannot meet the above requirements entirely. Moreover, as far as the authors know, there is no study for the real-time broadcast protocol to support the application-specific traffic model in WSN yet. Based on the above analysis, in this paper, we propose a new (*m*, *k*)-firm-based Real-time Broadcast Protocol (FRBP) by constructing a broadcast tree to satisfy the (*m*, *k*)-firm, which is applicable to the real-time model in resource-constrained WSNs. The broadcast tree in FRBP is constructed by the distance-based priority scheme, whereas energy efficiency is improved by selecting as few as nodes on a tree possible. To overcome the unstable network environment, the recovery scheme invokes rapid partial tree reconstruction in order to designate another node as the parent on a tree according to the measured (*m*, *k*)-firm real-time condition and local states monitoring. Finally, simulation results are given to demonstrate the superiority of FRBP compared to the existing schemes in terms of average deadline missing ratio, average throughput and energy consumption.

## 1. Introduction

Wireless sensor networks (WSNs) have been revolutionizing the way that people interact with the physical world by their diverse applications in different areas [1]. In an application-specific network like WSNs, applications are likely to need specific routing protocols according to the unique features of generated data [2]. For instance, emergency-detecting applications need real-time routing protocols, whereas state-monitoring applications require reliable routing protocols. Moreover, various types of communications such as unicast, multicast and broadcast also need to be supported according to the demands. In the case of fire alert application, for example, the detection of the fire may be delivered to a base station by the real-time unicast protocol, transmitted to fire fighters by the real-time multicast protocol and disseminated to all people in the local area for warning purposes by real-time broadcast protocols. In these scenarios, real-time communication is one of the areas highly focused on to support diverse and novel emerging sensor applications such as urgent data reporting and multimedia traffic management. However, supporting real-time communication is also a challenging issue in WSNs since the distinguishing features of WSNs as compared to traditional wired networks [3,4] lead to the building of harsh network environments that are composed of a large number of resource-constrained sensor nodes where their links are lossy and unstable. Therefore, WSNs naturally have dynamic environments such that states of nodes and links are easily changed. Moreover, the energy efficiency problem is another challenge to increase the network lifetime.

In terms of real-time communications, in general, hard real-time is impractical in WSNs due to the constrained resources and the dynamic network environment [5]. Thus, many studies propose soft real-time protocols that try to improve the probability of successful real-time delivery through probabilistic guarantee or heuristic algorithms. Soft real-time protocols, however, cannot guarantee the consistent Quality of Service (QoS) related to real time. Unlike typical approaches based on the hard and soft real-time model, recently, B. Li and K. Kim [6] suggest the (*m*, *k*)-firm-based Real-time Unicast Protocol (FRUP), which sets the criterion between hard real-time and soft real-time. The concept of (*m*, *k*)-firm real time is defined as at least *m* messages among any *k* consecutive messages have to meet their real-time constraint from a real-time data stream. This (*m*, *k*)-firm real-time model provides a more credible performance evaluation indicator than the simple probabilistic indicator, e.g., 80% real-time success ratio. Technically, FRUP tries to satisfy the (*m*, *k*)-firm real-time requirement by adjusting the data path in a way that reselects forwarding nodes by using two state indicators, Distance-Based Priority (DBP) and Local State Indicator (LSI). In addition to FRUP, since all other protocols for the (*m*, *k*)-firm real-time work are focused on one-to-one unicast, there is no existing multicast and broadcast protocol to support the (*m*, *k*)-firm model yet. Thus, (*m*, *k*)-firm based real-time unicast is required to be extended to multicast and network broadcast to apply this idea to various real-time applications.

To accommodate the above demands, in this paper, we deal with the (*m*, *k*)-firm-based real-time broadcast as the first step of the extension. From the point of view of efficiency, the current FRUP cannot be simply applied to network broadcast for following reasons. If data packets are delivered to each destination by FRUP in the case of network broadcasting (to all sensor nodes), many duplicated packet transmissions may lead to wasting of energy, increasing of transmission delay and congestion. Moreover, the algorithm to decide to rebroadcast in the original flooding and its variants need to be changed to meet the (*m*, *k*)-firm requirement. Based on these analyses, we set our goals, which are to support (*m*, *k*)-firm broadcast and improve energy efficiency.

To achieve these two goals, we propose a novel real-time network broadcast routing based on the (*m*, *k*)-firm real-time protocol, called the (*m*, *k*)-firm-based Real-time Broadcast Protocol (FRBP). The proposed scheme constructs a real-time broadcast tree by electing only some sensor nodes satisfying the real-time condition as intermediate transmission nodes to satisfy the first goal, the real-time constraint. Other sensor nodes, i.e., leaf nodes, only receive packets, but do not transmit them to other nodes for energy efficiency. Each sensor node can receive the data within the desired time deadline since each path (the source-intermediate node(s)-destination) over the tree that satisfies the real-time condition defined in SPEED [5]. The real-time broadcast tree also improves energy efficiency by avoiding network-wide flooding in which every sensor node has to transmit packets. To reduce energy consumption, the real-time broadcast tree is constructed in a way that includes as few intermediate nodes as possible. However, states of links and nodes can be changed anytime and anywhere due to the dynamic characteristics of WSNs. To deal with this unstable problem in the wireless link, the proposed scheme needs to include a recovery mechanism to deal with the state changes. For this, each intermediate node figures out local states such as node failure, link quality and real-time condition. Based on Distance-Based Priority (DBP) and local monitoring, the proposed scheme can avoid or recover real-time failures promptly by partial tree reconstruction. Moreover, the source node reconstructs the whole tree only in the worst case that local recovery schemes cannot guarantee the (*m*, *k*)-firm requirement. The performance of FRBP is evaluated by simulation scenarios in various conditions. Simulation results show that FRBP can satisfy the (*m*, *k*)-firm real-time requirement with reasonable energy consumption in various environments.

The rest of this paper is organized as follows. In Section 2, we summarize and analyze the related work. The details of FRBP are explained and described in the next section. The performance evaluation through simulation results is described in Section 4. Finally, the conclusion and further work are given in Section 5.

## 2. Related Work

To the best of our knowledge, the real-time approach for broadcast is not well explored yet. Therefore, this section summarizes related real-time protocols and broadcast protocols in WSNs. Since the proposed scheme uses geographic routing as the underlying routing protocol, we organize related work that is also based on geographic routing.

### 2.1. Real-Time Protocols

SPEED [5] is a well-known soft real-time unicast routing protocol that tries to probabilistically satisfy the real-time constraint in a hop-by-hop manner. It estimates the required transmission speed based on the desired time deadline and the end-to-end distance. Each intermediate forwarding node selects a next-hop node only among neighbors satisfying the required transmission speed. In other words, SPEED delivers a packet faster than the required transmission speed by forwarding the packet faster than the required transmission speed in every single hop. This concept is applicable in a wireless multi-hop network where the end-to-end delay is proportional to the physical distance the packet travels, unlike wired networks. Many studies propose variants of SPEED including FRUP and the proposed scheme in this work, FRBP. Multipath Multi-SPEED (MMSPEED) [7] is extended to provide a multipath and multilevel real-time routing protocol. Real-time Power-Aware Routing (RPAR) [8], Scalable Hierarchical Power Efficient Routing (SHPER) [9] and Power-Efficient Multimedia Routing (PEMuR) [10] raise the issue of energy efficiency with the real-time requirement in their respective fields. Fault-Tolerant SPEED (FT-SPEED) [11] deals with the void area problem of SPEED. In addition to unicast, Multicast Protocol for Real-time data Dissemination (MPRD) [12] proposes a real-time multicast protocol that constructs a novel multicast tree with the real-time constraint. Soft real-time protocols, however, cannot guarantee the consistent QoS related to the real-time requirement, but make their best effort. The consequent information of real-time delivery cannot be fed back to upstream nodes, and forwarding nodes discard packets when they do not find appropriate next-hop nodes.

**Figure 1:**
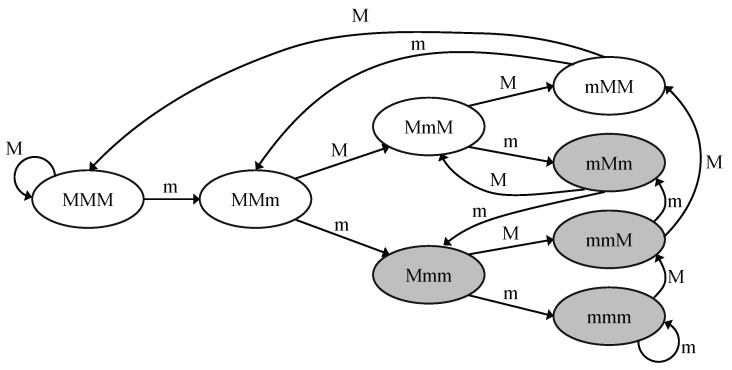
State transition diagram example of (2,3)-firm [6] where M and m represent deadline meet and miss, respectively.

The firm-based Real-Time (FRT) model [13] has similar features to both hard and soft real-time model in that it allows for infrequently missed deadlines while the system can survive task failures so long as they are adequately spaced. In the FRT model, the concept of (*m*, *k*)-firm real time is well defined, that is at least *m* messages among any *k* consecutive messages have to meet their real-time constraint from a real-time data stream. Figure 1 shows an example of state transition for the (2, 3)-firm real-time requirement. Each state represents that recent *k* messages meet or miss the deadline. For  example, MMm denotes the state where the most recent message missed its deadline and the two before it met their deadlines. With this state information, FRT mediates among multiple real-time streams. Each stream maintains the recent history, a state in Figure 1, and the system gives priorities to streams according to their states. If a stream is in a failing state (i.e., one of the shaded states in Figure 1), then a high priority is assigned to the stream.

FRUP [6] applies the (*m*, *k*)-firm model to real-time multi-hop unicast. In other words, FRUP uses the (*m*, *k*)-firm requirement to provide a consistent guarantee of real-time data delivery in multi-hop networks, whereas FRT uses it to mediate among multiple real-time streams in single-hop networks. FRUP tries to satisfy their (*m*, *k*)-firm real-time requirement by adjusting the data path, which reselects forwarding nodes by using two state indicators, DBP and LSI. DBP is an integer value that denotes the state of current real-time performance. A positive DBP value stands for meeting the (*m*, *k*)-firm requirement, while a negative DBP means the real-time failure state. DBP is calculated by the destination and fed back to upstream nodes and the source. FRUP also proposes LSI, which is calculated by each forwarding node to indicate the local condition of transmission. With two indicators, DBP and LSI, FRUP can perform a prompt fault recovery mechanism working at each forwarding node. FRUP however cannot be simply applied to network broadcast. If data packets are delivered to each destination by FRUP in the case of network broadcast (to all sensor nodes), many duplicated packet transmissions may lead to wasting of energy, increasing of transmission delay and congestion. Moreover, DBP feedback is impractical since DBP reporting messages from all sensor nodes also cause the same problems.

### 2.2. Broadcast Routing Protocols

To achieve broadcast, flooding [14] is a simple approach in that it provides fast broadcast even though it does not consider real-time requirements. The source node broadcasts a data packet, and then, the neighbors located within one hop from the source node receive the packet. Each neighbor rebroadcasts the packet to its own neighbors, but does not broadcast the same packet received before. This process is repeated until all sensor nodes receive the packet. Flooding is simple and fast since fast nodes are naturally selected to forward the packets. At each hop, a node receiving and processing the packet first can forward it immediately. However, frequent flooding can be a huge burden on the networks since it consumes many network resources. In flooding, all sensor nodes have to transmit the packet once and receive many duplicated packets from their all neighbors. Therefore, without any consideration for real-time service, this broadcast storm problem may lead to the increase of delay, collision and congestion, which adversely affect real-time performance.

As for energy-efficient broadcast, existing geographic broadcast protocols [15,16,17] try to minimize the number of forwarding nodes. Since the problem of minimizing the number of forwarding nodes is known to be NP-hard [18], they solve the problem by heuristic-based or topology-based plans. Sprinkler [15] reduces the number of forwarding nodes with the topology-based plan. It embeds a virtual grid structure over the network to separate sensor nodes in a suitable range for one-hop broadcast. Only one sensor node per cell becomes a forwarding node, and others only receive the packets. Moreover, construction of a virtual grid does not incur the transmitting and receiving costs since it is constructed by only the computation of each node. However, detouring paths of Sprinkler are a critical problem for real-time communication. In addition, V. Madhusudhana and S. P. Gururaj [16] decrease the number of forwarding nodes by a probability-based algorithm, one of the heuristic-based strategies. They made the observation that if the node has more siblings, there is a higher likelihood that its child nodes receive a packet, even if it does not forward the packet instantly. In this way, the more siblings a node has, the more its forwarding likelihood may be diminished. Meanwhile, if a node has numerous child nodes, it needs to forward the packet with a higher likelihood. Therefore, forwarding likelihood corresponds to the quantity of child nodes and conversely relative to the quantity of sibling nodes. S chakraborty et al. [17] propose the tree-based broadcast protocol, one of the topology-based plans. They construct a spanning tree out of all nodes in the network rooted at the source of the broadcast. The broadcast tree eventually becomes a Breadth-First Search (BFS) tree where each node maintains the shortest path to the root. This protocol also reduces the number of forwarding nodes. However, it is less effective than others since the first goal is the shortest path. As mentioned above, these broadcast protocols focus on energy efficiency, not the real-time requirement. In other words, they do not consider the node’s delay for electing forwarding nodes, so this can lead to real-time performance being unsatisfied.

In addition to the mentioned approach, there are some research works to improve the broadcast routing protocol by introducing the opportunistic flooding algorithm in WSN. First, S. Guo et al. [19] proposed opportunistic flooding in low-duty-cycle networks with unreliable wireless links and predetermined working schedules. Here, a new forwarder selection method is presented to resolve simultaneous forwarding operations through the hidden terminal problem and a link-quality-based backoff method. D. Chang et al. [20] present a probabilistic and opportunistic flooding algorithm that controls rebroadcasts and retransmissions opportunistically. The sender controls the number of retransmissions opportunistically according to error rate at its neighbors. Another interesting extension of opportunistic flooding is presented in [21]. They propose the contribution-level-based opportunistic flooding to achieve outstanding transmission efficiency and reliability to handle the failure case of the broadcast packet at the relay node. J. Luo et al. [22] address the network lifetime issue of the opportunistic flooding algorithm. Their approach called Energy Saving via Opportunistic Routing (ENS_OR) ensures minimum power cost during data relay and protects the nodes with relatively low residual energy. Moreover L. Zhang et al. [23] present a performance evaluation framework for the opportunistic flooding algorithm. They focus on two mechanisms based on acknowledgments and probabilistic retransmissions and analyze the behavior of the two mechanisms in different environments. Finally, Mayank Sharma et al. [24] present a comparative analysis for opportunistic routing in wireless sensor networks in terms of power usage, data aggregation, scalability and the data delivery model. These opportunistic broadcast protocols also do not consider real-time communication. Furthermore, opportunistic broadcast protocols tend to show longer delay than geographic broadcast protocols since they use the back-off delay plan for neighbor contention.

In wireless sensor networks, many communication protocols and applications rely on flooding for various networking purposes. Prior efforts focus on how to design efficient flooding algorithms; that is, they seek to achieve full reliability while reducing the number of redundant broadcasting across the network. To achieve efficient flooding, most of the existing protocols try to reduce the number of transmissions, which is decided without considering any online transmission result. In this paper, we propose a probabilistic and opportunistic flooding algorithm that controls rebroadcasts and retransmissions opportunistically. It seeks to achieve a target reliability required by an application. For this purpose, it makes a given node select only the subset of its one-hop neighbors to rebroadcast the same message. It considers node relations such as link error rates among nodes in selecting eligible neighbors to rebroadcast. The sender controls the number of retransmissions opportunistically by tracking the current status of message reception at its neighbors. Simulation is carried out to reveal that our proposed scheme achieves the given target reliability with less overhead than other flooding algorithms in most cases, thus prolonging the network lifetime.

## 3. Proposed Scheme: FRBP

To achieve two goals, real time and energy efficiency, the proposed scheme constructs a real-time broadcast tree to support the application-specific (*m*, *k*)-firm model. However, since states of links and nodes could be changed anytime and anywhere due to the dynamic characteristics of wireless sensor networks, the proposed scheme includes a recovery mechanism based on DBP and local monitoring to deal with state changes. The FRBP includes a procedure to construct and maintain the broadcast tree in a local and global way according to the current DBP value.

### 3.1. Real-Time Broadcast Tree Construction

The real-time broadcast tree is composed of three major components: root (source node), intermediate nodes and leaf nodes. To satisfy the (*m*, *k*)-firm real-time constraint, the tree construction scheme employs transmission speed estimation, which is the main idea of SPEED [5]. In other words, intermediate nodes in a tree are elected among sensor nodes satisfying the transmission speed. If all intermediate nodes are composed by nodes with a faster speed, then leaf nodes can receive data packets within the deadline consequentially. For energy efficiency, the tree construction scheme tries to elect as few intermediate nodes as possible. The more the leaf nodes, the less energy is consumed since leaf nodes, unlike intermediate nodes, only receive packets, but not transmit them to other nodes. The details are described as follows.

The source node generates a Real-time Broadcast Tree Construction (RBTC) message and disseminates it throughout the network by flooding [14]. The RBTC message includes source location, required transmission speed, sender location, sender transmission speed, sender’s parent location and hop count. The information is used for intermediate nodes’ election and recovery mechanism. The required transmission speed is calculated by the source node with the desired time deadline and the distance between the source node and the farthest destination. We assume that the source node is aware of the approximated network location since the application-specific property implies planned deployment. The source node measures the farthest network edge from itself as the farthest destination. The hop count means the number of hop counts from the source node and also the level of the broadcast tree.

**Figure 2:**
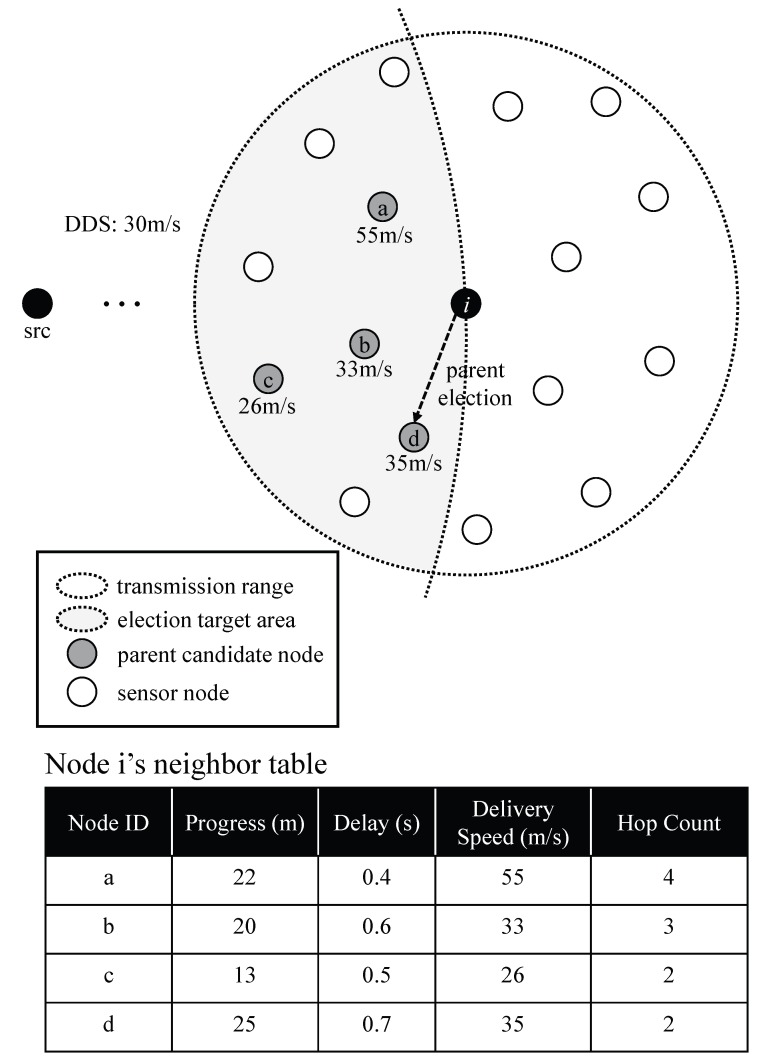
An example of parent election through Desired Delivery Speed (DDS).

Each sensor node elects its own parent within the neighbors with the election rules related to the tree construction goals. After a node receives the RBTC message, the node performs the parent election process. As described in Algorithm 1, the node excludes neighbors located farther than itself from the source node from being parent candidates. The node also excludes neighbors that do not satisfy the transmission speed for real-time data dissemination. All remaining neighbors could be parent candidates since they satisfy the real-time condition. The node then elects a parent among the candidates with additional rules for energy efficiency. The node compares the hop counts and holds candidates having the least hop count. Lastly, the furthest candidate from the source node is elected as a parent node. These last two rules could contribute to reducing the number of intermediate nodes. Figure 2 shows an illustration of the process. After the parent election process is completed, child nodes announce child information to their parents. Unelected sensor nodes become leaf nodes and do not participate in the real-time broadcast. Each node maintains the information of neighbors for recovery mechanism, even though the neighbors are eliminated from being parent candidates.

The real-time data packets are disseminated along the real-time broadcast tree. The packets could arrive at all sensor nodes since every intermediate node satisfies the required transmission speed. The real-time broadcast tree could reduce energy consumption in comparison with flooding since leaf nodes do not transmit packets, unlike flooding where all nodes transmit packets.

**Algorithm 1** Real-time broadcasting tree construction
1:Dist(i,j): distance between node *i* and *j*2:SingleHopDelay(i,j): single hop delay during data transmission from *i* to *j*3:DDS: desired delivery speed4:$NS_{i}$: neighbors’ set of node *i*5:$PCS_{i}\leftarrow \{n\in NS_{i}|Dist\left(src,i\right)-Dist\left(src,n\right)>0\}\;▹$ parent candidate set closer to a source than node *i*6:$PCSRB_{i}\leftarrow \{j\in PCS_{i}|\frac{Dist(i,j)}{SingleHopDelay(i,j)}\geq DDS\}\;▹$ parent candidate set for real-time broadcasting7:**if** $PCSRB_{i}==\mathrm{then}\;▹$ if node *i* has no candidate satisfying a desired speed8:  $parentNode_{i}\leftarrow \underset{j\in PCS_{i}}{\arg\;\max}\{\frac{Dist(i,j)}{SingleHopDelay(i,j)}\}\;▹$ designate a parent node having the maximum delivery speed9:**end** **if**10:$T\leftarrow \{t\in PCSRB_{i}|min\left(HopCount\left(t\right)\right)\}$11:$parentNode_{i}\leftarrow \underset{t\in T}{\arg\;\max}\left\{Dist\left(src,t\right)\right\}$


### 3.2. Data Transmission through the Real-Time Broadcast Tree

The header of each data packet includes the following information: source node information, sequence number, path information, sender ID, delay information of a sender and location information of a sender. The sequence number of the data prevents duplicated reception by comparing the value in the cache. Delay information and location information of a sender are used for delivery speed estimation.

Fundamentally, when a source node generates a data packet, the data are forwarded by broadcast. Neighbor nodes of a sender listen to the data and match up the ID of the sender with their parents’ ID. If the IDs are matched, corresponding neighbor nodes rebroadcast the data. Then, the data packet is spread to all nodes downstream of the pre-constructed real-time broadcast tree. To measure the (*m*, *k*)-firm requirement, each node maintains DBP and real-time transmission information at each data transmission for local monitoring as presented in Equation (1).

(1) $$DBP_{sd}=k_{s}-m_{s}-n\left(s_{sd}\right),$$

where $DBP_{sd}$ is the measured DBP value of stream *s* at destination *d*, $k_{s}$ and $m_{s}$ come from the (*m*, *k*)-firm real-time requirement of stream *s*, $s_{sd}$ denotes the current (*m*, *k*)-firm state of stream *s* at destination d and $n(s_{sd})$ means the number of packets missing their deadline in state $s_{sd}$. The (*m*, *k*)-firm state is mentioned above in Figure 1. A positive DBP value, including zero, stands for meeting the (*m*, *k*)-firm requirement, while a negative DBP means the real-time failure state.

When a child node receives data, the node calculates a delivery speed by a sender’s delay and distance between the sender and each child. In real-time broadcast, since every sensor node is a destination, all of them need to maintain their own DBP value. If the measured speed exceeds the real-time delivery constraint, it increases the DBP value of the child node. Otherwise, the node decreases the DBP value. According to the result of the DBP value update, the node determines fault reporting upstream. Furthermore, intermediate nodes have the responsibility to support a series of child nodes downstream, and thus, the node deals with delivery speed for each transmission even if its own DBP status is fine. The detailed concerns about the local monitoring will be discussed in the next subsection.

### 3.3. Local Real-Time Failure Avoidance and Recovery

Subtree or whole tree reconstruction naturally incurs a high cost. Thus, intermediate nodes avoid real-time broadcast faults by local monitoring. In this section, we describe the local avoidance and recovery mechanism to deal with this issue.

In firm-based real-time routing, destinations maintain their DBPs with the history of success or failure of real-time data delivery. firm real-time unicast maintains DBP at sources and destinations, and forwarding nodes on the paths between both are aware of the related information for real-time data delivery. Meanwhile, in real-time broadcasting, each destination, i.e., each sensor node in the case of network broadcast, maintains its own DBP. That is to say, it is possible to monitor the surroundings at all times. Thus, if DBP values are negative, the relevant node starts the fault recovery process. In the tree structure, real-time failures of intermediate nodes could lead to a number of failures downstream. Intermediate real-time failures are caused by node failure, link failure and increased delay at their parents. In the last case, even though the intermediate node receives data packets within the deadline, it could lead to real-time failures of downstream nodes. Intermediate nodes therefore monitor local states related to real-time data delivery including their parents. Note that intermediate nodes cannot obtain DBPs from downstream nodes in the normal case.

An intermediate node regards its parent as real-time failure in three cases: (i) it does not receive a periodic beacon from the parent; (ii) it does not receive the data packet; and (iii) the transmission speed of the parent becomes lower than the required transmission speed. If a parent node is temporarily down or occupied by a full data packet queue due to congestion, it could not send a beacon or data. However, since this kind of failure might be recovered shortly, the child nodes replace the parent with the second-best node in their parent candidate set until the parent becomes stable. If the parent is permanently depleted, the second-best node continues its parent role.

The transmission speed of the parent could be obtained from both the beacon message and the header of the data packet. The intermediate node then re-elects another parent. The reelection rules are exactly the same as the parent election rules in real-time broadcast tree construction. If the re-elected parent is a leaf node, the parent operates as an intermediate node after election. The new parent may not cover all leaf nodes of the previous parent, but this problem could be solved by DBP-based local failure recovery. This local monitoring and recovery process by intermediate nodes could result in real-time failure avoidance or resilient real-time failure recovery on the condition that intermediate nodes cannot obtain DBPs of downstream nodes.

When this avoidance mechanism works normally, the DBP value of each node could maintain a fine condition. Though the value is shown to be negative, it would return to the normal condition by substitution of intermediate nodes. Only in the case of an abnormal state is which there is no qualified nodes for substitution parents or some area suffers from bad conditions due to the congestion repeats after the repetition of fault avoidance, DBP values would be worse. That is to say, if the avoidance mechanism works properly, such situations would rarely occur.

Since leaf nodes have no child nodes, they focus on their own real-time data reception. Thus, they measure only their own DBP, and the fault avoidance mechanism works based on DBP. In the case of a DBP value falling down to a negative one, a relevant leaf node designates a parent as in the case of intermediate nodes. However, since it might recover shortly after due to the DBP characteristics, it causes an unnecessary reporting process whenever the values are negative. When a leaf node shows temporal fluctuation, DBP reflects its historical characteristics, and we expect that will be recovered after a while. As discussed above, since network-wide broadcast by DBP reporting causes severe energy consumption, the reporting process should be kept to a minimum. In this paper, we assume that the report condition of node *i* is $DBP_{i}<\left[-m/2\right]$. Algorithm 2 shows the entire procedure for failure avoidance at each node.

At this point, each intermediate node also determines whether it uploads received DBP-Based Fault Reporting (DBFR) messages through upstream by policies of a parent. In this paper, we assume that intermediate nodes upload DBFR even if one DBFR is received. In order to prevent reporting excessive DBFR messages, each intermediate node uploads aggregated DBFR toward a source.

Through the described procedure, the source could be aware of how many DBP faults occur. The source also expects flexible recovery through the avoidance because of the high cost of tree reconstruction. In the meantime, when the number of DBFR message exceeds a predefined threshold, broadcasting tree reconstruction might be conducted according to administration policies. Namely, the source regards such a situation as unsatisfactory for the goal of real-time broadcasting and performs the tree reconstruction. Additionally, if the reconstruction is frequently performed, it is considered that the entire network could not provide real-time broadcasting capacity; though in such bad circumstances, data delivery is needed, it temporarily runs the flooding method.

**Algorithm 2** Real-time failure avoidance
1:$PCSRB_{i}$: parent candidate set for real-time broadcasting2:$DBP_{i}$: DBP value of node *i*3:DDS: desired delivery speed4:DS: delivery speed between two nodes5:**if** $parentNode_{i}\;==\;\mathrm{then}\;▹$ if a current parent node of *i* is in the case of node or link failure6:  $T\leftarrow \{t\in PCSRB_{i}|min\left(HopCount\left(t\right)\right)\}$7:  $parentNode_{i}\leftarrow \underset{j\in T}{\arg\;\max}\left\{Dist\left(src,j\right)\right\}$8:**else if** $DeliverySpeed(i,parentNode_{i})<DDS\;\mathrm{then}\;▹$ if a current parent node of *i* is in the case of bad DS9:  **if** $numChildNode_{i}>0\;\mathrm{then}\;▹$ if node *i* is an intermediate node10:   $T\leftarrow \{t\in PCSRB_{i}|DBP_{t}\geq 0\}$11:   **if** T== **then**12:    $T\leftarrow \{t\in PCSRB_{i}\}$13:    **if** T== **then**14:     $T\leftarrow \{t\in PCS_{i}|DBP_{t}\geq 0\}$15:    **end if**16:   **end if**17:   $parentNode_{i}\leftarrow \;\underset{j\in T,j\neq parentNode_{i}}{\arg\;\max}\{DeliverySpeed\left(i,j\right)\}\;▹$ the second best node18:  else               ▹ if node *i* is a leaf node19:   $T\leftarrow \{t\in PCSRB_{i}|DBP_{t}\geq 0\}$20:   **if** T== **then**21:    $T\leftarrow \{t\in PCS_{i}|DBP_{t}\geq 0\}$22:    **if** T== **then**23:     $T\leftarrow \{t\in PCSRB_{i}\}$24:    **end if**25:   **end if**26:   $parentNode_{i}\leftarrow \underset{j\in T,j\neq parentNode_{i}}{\arg\;\max}\{DeliverySpeed\left(i,j\right)\}\;▹$ the second best node27:  **end if**28:**else if** $DBP_{i}<\lfloor \frac{k-m}{2}\rfloor \;\mathrm{then}\;▹$ if the DBP value of node *i* is poor29:  $T\leftarrow \{t\in PCSRB_{i}|DBP_{t}\geq 0\}$30:  $parentNode_{i}\leftarrow \underset{j\in T,j\neq parentNode_{i}}{\arg\;\max}\{DeliverySpeed\left(i,j\right)\}\;▹$ the second best node31:**end** **if**


## 4. Performance Evaluation

In this section, we compare the performance of the proposed FRBP with four other protocols: flooding [14], Sprinkler [15], the Probability-based Energy-efficient Broadcast Protocol (PEBP) [16] and the Opportunistic Energy-efficient Broadcast Protocol (OEBP) [21]. For the performance evaluation, we do not use FRUP due to the wide gap between the unicast and broadcast protocol. This implies that real-time data delivery to all sensor nodes by FRUP is still unreasonable in WSNs. Since there was no previous real-time broadcast protocol, four target protocols are chosen among the broadcast protocols. They are known as the most suitable broadcast protocols to compare with FRBP for the two goals, real-time broadcast and energy efficiency. Flooding ideally broadcasts packets more rapidly, whereas Sprinkler is a more energy-efficient broadcast protocol. PEBP and OEBP are the most recent broadcast protocols based on geographic routing and opportunistic routing, respectively.

These protocols were implemented in NS-3 [25], one of the widely-used open source network simulators in the research community. We deploy 1000 sensor nodes in a 500 m × 500 m area, and then, each sensor has several neighbors probabilistically. Sensor nodes follow the specification of MICA2 [26]. We conduct 20 rounds to obtain average result values. In each round, a source is randomly determined among sensor nodes, and the source sends data 80 turns for generating the data stream. We set a desired delivery time deadline to 10 s, and thus, a desired delivery speed is derived at 70 m/s to cover the whole network area. The experiments are conducted under the (4, 5)-firm real-time requirement. Furthermore, average hop delay is set to 0.4 s, and the delay is randomly determined with the values range of ± 0.2 s. The delay varies with the random value in the range mentioned earlier and the random period (from 1–10 turns). Each link and each node have failed in every turn with a low probability, 0.5%. Unlike node failures, sensor nodes recover from link failures after random turns. Summarized simulation parameters are depicted in Table 1.

The Deadline Missed Ratio (DMR) is the ratio of the number of hitting the (*m*, *k*)-firm real-time requirement to the number of data packets delivered for a destination. Average DMR (ADMR) therefore means the average value of deadline missed ratios for all destinations. Energy consumption means the total energy consumed by all sensor nodes during one round of simulation. Throughput is the number of received Packets Per Second (PPS) for a destination. Average throughput means the Average value of BPS (APPS) for all destinations. Each destination starts calculating BPS when it receives the first packet. The three factors are the average values of 20 rounds of simulation.

**Figure 3:**
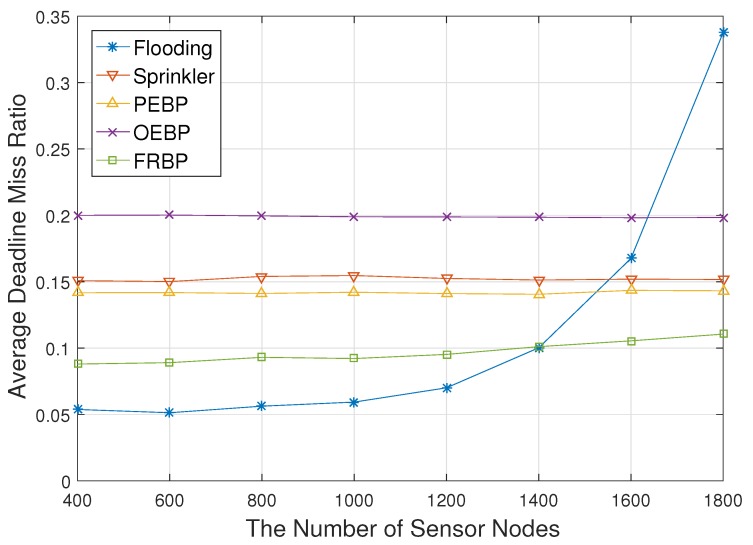
Deadline missed ratio impacted by the number of nodes. PEBP, Probability-based Energy-efficient Broadcast Protocol; OEBP, Opportunistic EBP; FRBP, (*m*, *k*)-firm-based Real-time Broadcast Protocol.

Figure 3 shows the ADMR with respect to the number of sensor nodes. The increment of the number of sensor nodes means the increment of the number of destinations in network broadcast. The number of sensor nodes also indicates the density since the area of the network is fixed. Flooding shows better performance than others, but ADMR exponentially increases as the number of sensor nodes increases. In flooding, high density leads to collisions and congestions since all sensor nodes have to transmit packets. This problem consequently causes the increase of hop delay and real-time failures. Other protocols are nearly impervious to the number of sensor nodes because they just select proper next-hop nodes in each path regardless of the number of neighbors. The ADMR of OEBP shows the highest value due to the back-off delay for neighbor contention. The ADMR of Sprinkler is a little higher than the PEBP due to the detouring paths. Note that the end-to-end delay is proportional to the physical distance the packet travels in a wireless multi-hop network. PEBP, OEBP and Sprinkler show worse performance than FRBP, the proposed protocol, since they do not consider the node’s delay for electing forwarding nodes. The ADMR of FRBP slightly increases due to the increase of signaling messages for the tree construction and fault recovery. The amount of increase however is much less than flooding.

**Figure 4:**
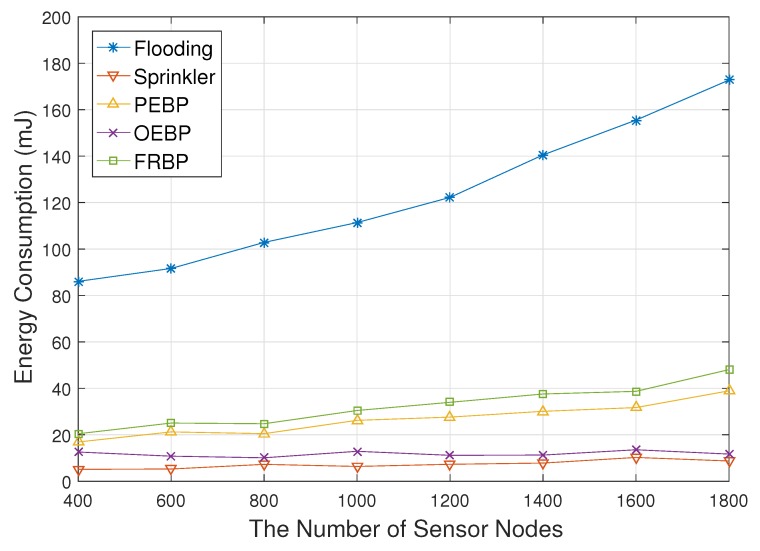
Average energy consumption impacted by the number of nodes.

Figure 4 represents the energy consumption depending on the number of sensor nodes. We do not take beacon signaling into account since it is the same among the five protocols. Flooding consumes much more energy than comparative protocols since all sensor nodes have to transmit packets and each node has to receive packets from all neighbors even though it discards most packets. OEBP and Sprinckler consume less energy than others since they do not need to generate any signaling messages for topology construction and recovery. They are also unaffected by the density level for the same reason. FRBP consumes a little more energy than PEBP due to the recovery process for real-time failure. PEBP has a recovery process for communication failure, but does not recover real-time failure. The number of forwarding nodes of FRBP is unaffected by the density level since neighbors of an intermediate node become leaf nodes, which do not forward data packets. The energy consumption of FRBP however slightly increases due to the increase of signaling messages for the tree construction and fault recovery process.

**Figure 5:**
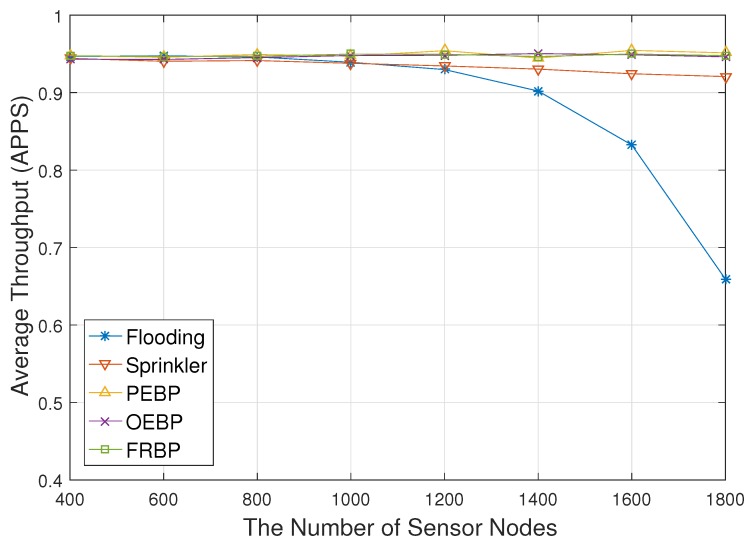
Average throughput impacted by the number of nodes.

Figure 5 shows the average throughput depending on the number of sensor nodes. The graph pattern is similar to the opposite shape of Figure 3. The average throughput of flooding exponentially decreases as the number of sensor nodes increases because high density leads to critical packet loss by collisions and congestions in flooding. This figure shows that decrease of throughput (i.e., increase of packet loss) leads to the decrease of real-time performance.

**Figure 6:**
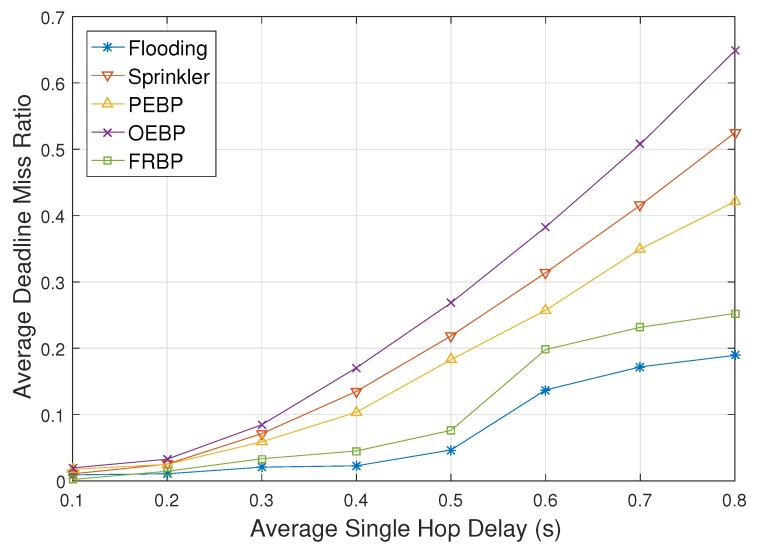
Deadline missed ratio impacted by single hop delay.

Figure 6 illustrates the ADMR with respect to the average single hop delay. The increase of hop delay means the degradation of the network environment, which leads to failure of delivery within the deadline. The ADMR of OEBP, PEBP and Sprinkler increases more than the other two protocols according to the increase of average single hop delay. Since they do not consider the real-time constraint, the probability of electing time-consuming nodes increases as average single hop delay increases. The ADMR of Sprinkler is higher than that of PEBP due to the detouring paths, and the ADMR of OEBP is higher than that of PEBP due to the back-off delay for neighbor election. After the average single hop delay meets 0.5, flooding and FRBP have difficulty, and their ADMR increases rapidly. When the network environment gets worse, the furthest destinations may fail to receive packets within the time deadline at first. In the case of flooding and FRBP, the further the location from the source, the greater the number of destinations. The gradients of increase therefore grow less as the average single hop delay increases more.

**Figure 7:**
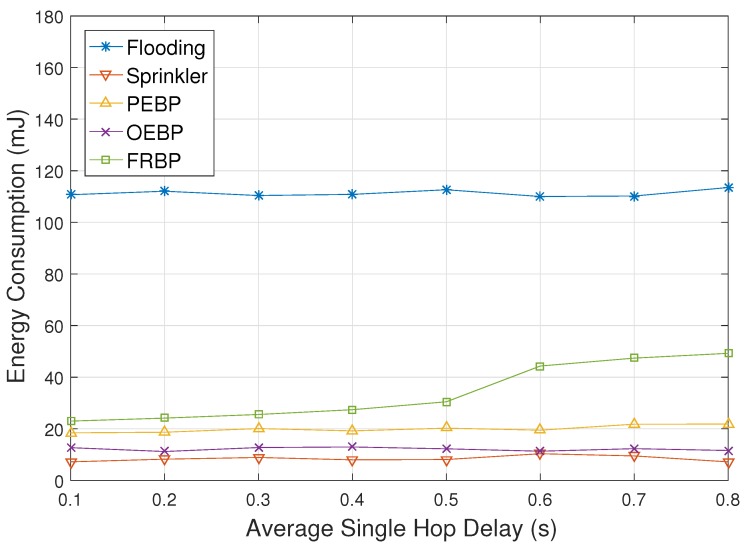
Energy consumption impacted by single hop delay.

Figure 7 represents the energy consumption depending on the average single hop delay. It is certain that flooding consumes much energy. Four protocols except FRBP are nearly impervious to the hop delay since they do not perform any operation against real-time failure. The energy consumption of FRBP however slightly increases as hop delay increases especially after 0.5 due to the fault avoidance and recovery process.

**Figure 8:**
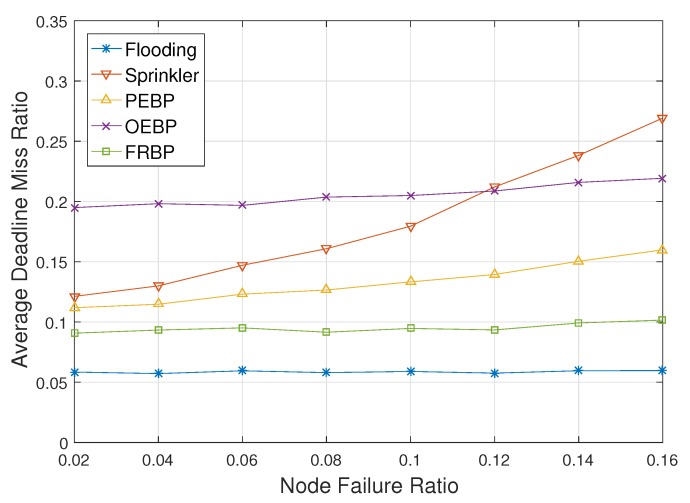
Deadline missed ratio impacted by the node failure ratio.

Figure 8 shows the ADMR with respect to the node failure ratio. In simulation, each node has failed in every turn with a low probability and does not recover since node failure is primarily caused by exhaustion of energy and physical destruction. After a node has failed, the DMR of the node is not included in ADMR. As the node failure ratio increases, the ADMR of Sprinkler increases exponentially. The failures of non-forwarding nodes do not affect ADMR, but the failure of a forwarding node leads to consecutive real-time failures of downstream nodes. The consecutive failures moreover do not recover. PEBP has same problems, but could probabilistically recover the situation. PEBP therefore shows better performance than Sprinkler. FRBP recovers the failures of intermediate nodes, as well as downstream nodes by local real-time failure avoidance. FRBP recognizes a failed node as a node that does not satisfy the real-time constraint. Therefore, the ADMR of FRBP marginally increases, but is not critically affected by the node failures. Flooding is nearly impervious to the node failure ratio since it does not elect forwarding nodes, and every node forwards packets. Even when a node has failed, its neighbors can receive packets from other nodes. OEBP is also unaffected by the node failure since forwarding nodes are elected among neighbors that actually receive the packet. A failed node has no chance of being elected. However, OEBP still shows worse performance than the others due to the election process.

**Figure 9:**
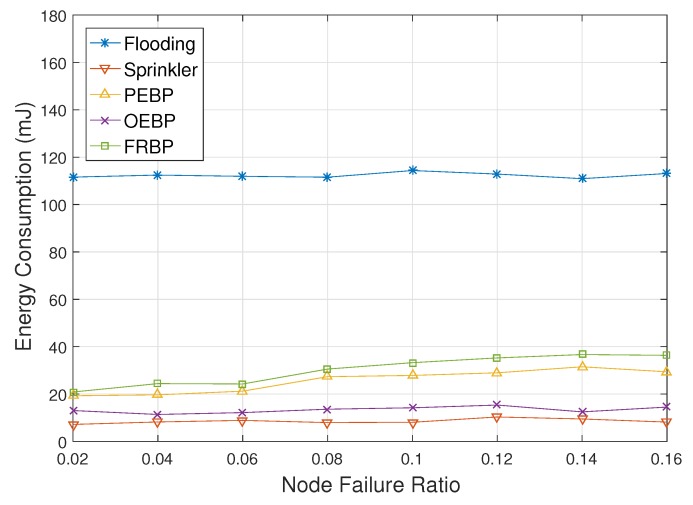
Average energy consumption impacted by the node failure ratio.

Figure 9 represents the energy consumption depending on the node failure ratio. Flooding, Sprinkler and OEBP are almost unaffected by the node failure ratio since Sprinkler does not have any recovery process, and flooding and OEBP do not need it. The energy consumption of Sprinkler rather decreases slightly since downstream nodes of a failed node stop forwarding packets. Against node failures, PEBP and FRBP perform local failure recovery processes that cause energy consumption for signaling. The energy consumption of PEBP and FRBP therefore increases according to the increase of the node failure ratio, but signaling cost is much lower compared with data delivery cost. PEBP consumes a little more energy than PEBP since it performs the recovery process against not only node failures, but also real-time failures.

**Figure 10:**
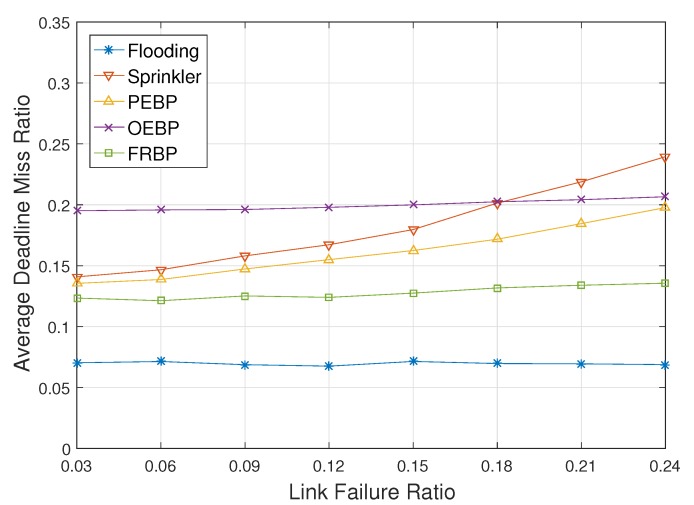
Deadline missed ratio impacted by the link failure ratio.

**Figure 11:**
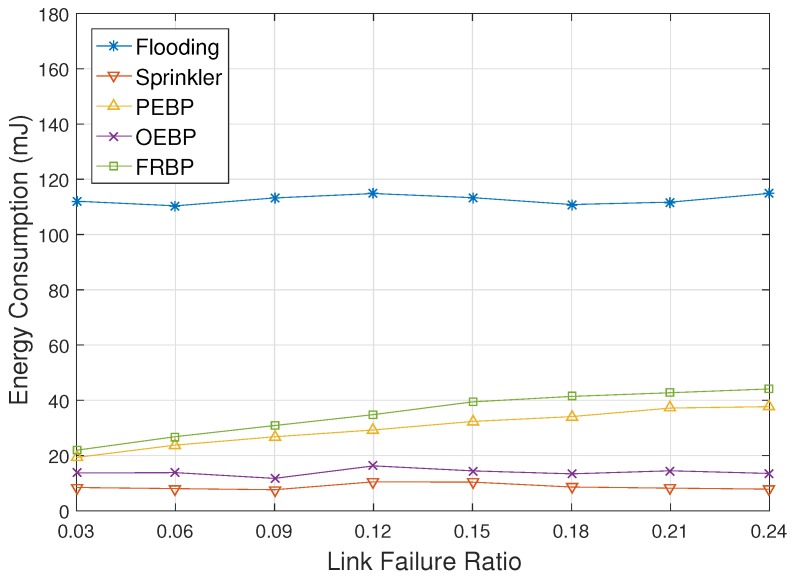
Average energy consumption impacted by the link failure ratio.

Figure 10 and Figure 11 show ADMR and energy consumption with respect to the link failure ratio, respectively. Unlike node failures, link failures are recovered since they are caused by temporary troubles. The two graphs show a similar pattern as shown in Figure 8 and Figure 9. The increase of ADMR in Sprinkler however is a little more gradual than Figure 8 due to the recoveries of link failures. Figure 8 also shows that FRBP can handle the link failures, as well as node failures in an efficient way.

According to the performance evaluation, our main observations are as follows. Flooding is generally efficient for real-time broadcasting except dense networks, but uses too much energy. Broadcast storming is also a well-known problem of flooding. The proposed protocol, FRBP, has better performance for real-time broadcasting than the other three protocols in various situations. FRBP consumes a little more energy than the three protocols, but we consider that this is reasonable; because the other protocols do not consider the real-time constraint and do not have any avoidance or recovery processes for real-time failures.

## 5. Conclusions and Further Work

In this paper, we proposed FRBP, a real-time network broadcast protocol for the (*m*, *k*)-firm real-time model, that is applicable in WSNs. FRBP operates over the real-time broadcast tree that consists of only some sensor nodes satisfying the real-time condition as intermediate transmission nodes for the real-time constraint. In the broadcast tree, leaf nodes only receive packets, but do not transmit them for energy efficiency. While constructing the broadcast tree, FRBP considers two major inherent characteristics of WSNs, resource-constrained sensor nodes and an unstable network environment. For resource-constrained sensor nodes, FRBP tries to elect as few intermediate nodes as possible in the tree. To overcome the unstable network environment, FRBP performs local real-time failure avoidance and recovery based on DBP by measuring (*m*, *k*)-firm real-time conditions, as well as monitoring local states. Finally, simulation results demonstrated that FRBP can satisfy the (*m*, *k*)-firm real-time requirement with reasonable energy consumption in various environments. In summary, FRBP consumes on average 26% the energy of flooding, and its deadline missed ratio is on average 58% that of the other three protocols.

The remaining further work will be focusing on extending FRBP to support multicast as group communications. In addition, practical applications such as real-time mobile event tracking and its deployment issues will be studied. Moreover, extensive simulation studies will be conducted by analyzing the impact of multiple (*m*, *k*)-firm streams.

## Figures and Tables

**Table 1:** Summary of simulation parameters.

Parameter	Value
Simulator	NS-3
Transmission range	30 m
MAC layer	IEEE 802.11 b
Network size	500 m × 500 m
The default number of nodes	1000
Transmitting/receiving power	3.12 μJ/2.34 μJ
Desired delivery time deadline	10 s
Desired delivery speed	70 m/s
Real-time requirement	(4, 5)-firm
The number of simulation rounds	20
The number of packets in a round	80
Packet generation rate	Constant Bit Rate (CBR), 1 packet/s
